# Proteome Analysis of Nasolacrimal Duct Lavage Fluid in Patients with Primary Acquired Nasolacrimal Duct Obstruction

**DOI:** 10.3390/ijms27062616

**Published:** 2026-03-12

**Authors:** Heejeong You, Wonseok Bang, Byeongsoo Kang, Seunghoon Back, Junyoung Park, Minjung Ju, Jong-Moon Park, Helen Lew

**Affiliations:** 1 Department of Ophthalmology, Bundang CHA Medical Center, CHA University, Seongnam-si 13496, Republic of Korea; hjyoueye@gmail.com; 2 Basil Biotech, Incheon 21984, Republic of Korea; wsbang@basilbiotech.com (W.B.);; 3 College of Pharmacy, Gachon University, Incheon 21936, Republic of Korea; 4 Department of Chemistry and Center for Proteogenome Research, Korea University, Seoul 02843, Republic of Korea; likeone61@korea.ac.kr

**Keywords:** PANDO, epiphora, nasolacrimal duct irrigation, tear proteomics

## Abstract

Primary acquired nasolacrimal duct obstruction (PANDO) is a common cause of epiphora in adults, yet the biochemical environment within the nasolacrimal duct (NLD) remains poorly understood. This study aimed to characterize the proteomic composition of NLD lavage fluid and identify subtype-specific molecular features distinguishing membranous and mucinous obstruction. Paired tear and NLD lavage fluid (NLD-LF) samples were collected from patients undergoing dacryoendoscopic recanalization, and proteomic profiling was performed using LC–MS/MS. A total of 1345 proteins were identified in NLD-LF and 767 in tear fluid, revealing a distinct NLD-specific proteome. Although the membranous and mucinous subtypes shared broadly similar protein compositions, differentially expressed proteins highlighted divergent biochemical pathways. The membranous subtype showed enrichment of keratinization-related processes involving *KRT1*, *KRT9*, and *KLK13*, suggesting epithelial remodeling and cornification. In contrast, the mucinous subtype exhibited upregulation of proteins involved in lipid metabolism, carboxylic acid biosynthesis, and sulfur compound metabolism, including *ALOX15B*, *LCAT*, and *GSTM4*, indicating metabolic conditions that promote mucin–lipid interactions, glycan sulfation, and redox-dependent mucin cross-linking. These findings provide new insights into the protein composition of NLD lavage fluid and suggest molecular differences between the membranous and mucinous obstruction subtypes.

## 1. Introduction

Although primary acquired nasolacrimal duct obstruction (PANDO) is among the most common causes of adult epiphora, its precise etiology remains unclear. Multiple hypotheses have been proposed, and recent reviews have outlined its etiopathogenesis as a multifactorial process involving both local and systemic factors [1].

Recent studies have reported that alterations in tear proteins may act as possible contributors to the pathogenesis. Elevated levels of pro-inflammatory mediators such as interleukin (IL)-6, IL-8, and tumor necrosis factor-α (TNF-α) have been demonstrated in the tears of patients with nasolacrimal duct obstruction compared with healthy controls, suggesting that chronic inflammation and mucosal remodeling play important roles in the disease process [2,3,4].

To further extend cytokine-based studies, we previously performed a comprehensive proteomic analysis of the tear fluid [5]. Through this study, we identified differences in protein expression between PANDO patients and healthy controls, and further observed that even among PANDO patients, protein expression profiles differed between the mucinous-type and membranous-type subgroups.

However, tear fluid, which is collected from the ocular surface, has its limitations to reflect the local biochemical environment within the nasolacrimal duct (NLD) itself. To address this limitation, we collected nasolacrimal duct lavage fluid (NLD-LF) during dacryoendoscopy-assisted recanalization procedures and performed LC–MS/MS–based proteomic analyses of tear fluid and NLD-LF collected from patients with PANDO.

In this study, we aim to study proteomic differences between tear and NLD lavage fluid and compare protein composition of NLD lavage fluid based on the types of obstruction.

## 2. Results

### 2.1. Clinical Characteristics of the Study

A total of 15 patients were enrolled in this study, including 9 with the membranous-type obstruction and 6 with the mucinous-type obstruction. There were no significant differences in age, sex, or preoperative tear meniscus height between the two groups (Table 1). Although a higher proportion of patients in the membranous group showed complete obstruction on dacryocystography, the overall distribution of dacryocystographic findings did not differ significantly between the membranous and mucinous groups. Comparisons between the membranous-type and mucinous-type groups were conducted using the Mann–Whitney U test and the chi-square test.

### 2.2. Global Proteome Profiling of NLD Fluid and Tear Fluid

**Figure 1:**
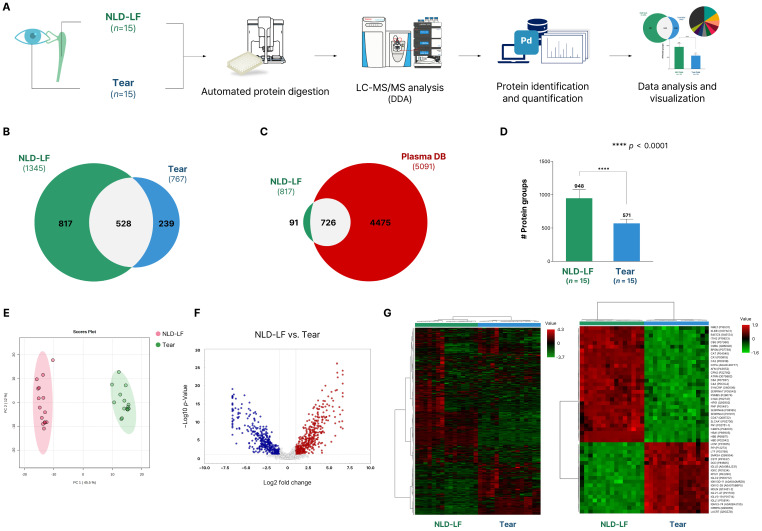
Overview of the proteome profiling of NLD-LF and tear. (**A**) Schematic workflow of proteomic analysis of NLD-LF and tear. (**B**) Comparison of identified proteins between NLD-LF and tear. (**C**) Comparison of 817 NLD-specific proteins, which were not detected in tear, with the human plasma proteome database. (**D**) Comparison of the number of average identified proteins in NLD-LF and tear. (**E**) Principal component analysis (PCA) of proteomic profiles. (**F**) Volcano plot illustrating relative abundance trends between NLD-LF and tear proteomes (non-quantitative comparison). Red dots indicate upregulated proteins in NLD-LF, and blue dots indicate downregulated proteins. (**G**) Hierarchical clustering of normalized protein abundances in NLD-LF and tear, showing the total proteome (**left**) and the top 50 most significantly differentially expressed proteins based on *p*-Value from *t*-test (**right**).

To investigate the differences in protein composition between NLD-LF and tear fluid, LC–MS/MS–based proteome analysis was conducted on paired samples collected from the same patients (Figure 1A).

A total of 1345 proteins were identified in NLD-LF (*n* = 15) and 767 proteins in tear fluid (*n* = 15), of which 528 proteins (33.3%) were commonly detected in both sample types (Table S1). In contrast, 817 proteins were uniquely identified in NLD-LF and 239 proteins were specific to tear fluid, indicating distinct proteomic profiles between the two fluids (Figure 1B).

To further exclude the potential influence of tear fluid or blood-derived proteins in the NLD-LF samples and to identify proteins specific to NLD-LF, we compared our dataset with previously reported human plasma proteome data [6]. This analysis revealed 91 proteins, defined as the NLD-specific proteome, that were detected exclusively in NLD-LF and were absent from both tear fluid and the human plasma proteome (Figure 1C).

A comparison of the average number of identified proteins across sample types showed that NLD-LF contained an average of 948 proteins, whereas tear fluid contained 571 proteins (Figure 1D). This corresponds to approximately 1.7-fold more proteins detected in NLD-LF than in tear fluid, and the difference was statistically significant.

Principal component analysis (PCA) revealed a clear separation between NLD-LF and tear fluid based on their proteomic profiles (Figure 1E). The first principal component (PC1) accounted for 45.5% of the total variance and distinctly separated the two groups, while the second principal component (PC2) explained 12.9% of the variance and reflected within-group variability. Although the matrix properties of NLD-LF and tear fluid differ, which should be considered when interpreting quantitative differences, the analysis of protein expression levels revealed numerous proteins with differential abundance between the two sample types (Figure 1F). Furthermore, heatmap analysis using all identified proteins and the top 50 DEPs revealed robust and coherent clustering of NLD-LF and tear fluid, with distinct separation between the two groups and opposing expression trends among the top DEPs (Figure 1G). This clustering pattern was consistent with the PCA results and reflects distinct protein expression profiles between NLD-LF and tear fluid.

### 2.3. Comparative Proteome Analysis in NLD-LF, Tear Fluid, and NLD-Specific Proteome

A total of 1345 and 767 proteins identified from NLD-LF and tear fluid, respectively, along with 91 NLD-specific proteins that were not detected in either tear fluid or the human plasma proteome, were used to select the top 10 most abundant proteins for each group (Table 2).

In NLD-LF, the top 10 proteins included several blood-associated proteins such as *HBA1*, *HBB*, and *ALB*, as well as proteins related to complement components (C3) and redox-associated proteins (*PRDX2*, *CAT*, *HPX*). *MUC5AC* was also among the highly abundant proteins in NLD-LF.

In tear fluid, the top 10 proteins corresponded to previously reported major tear proteins, including *LYZ*, *LTF*, and *LCN1* [7]. Additional highly abundant proteins were associated with epithelial structural components (*KRT1*, *KRT9*, *KRT10*) and secreted proteins such as *IGHA1* and *LACRT*.

Among the NLD-specific proteins, highly expressed proteins were involved in structural organization (*TUBA1A*, *CYFIP2*, *TSPAN1*), intracellular trafficking and secretion (*HPS1*, *SPATA1*), oxidative metabolism (*ALDH3B1*, *ALOX15*), and energy-related processes (*NME1–NME2*).

### 2.4. Comparative Functional Analysis of NLD-LF, Tear Fluid, and NLD-Specific Proteomes

**Figure 2:**
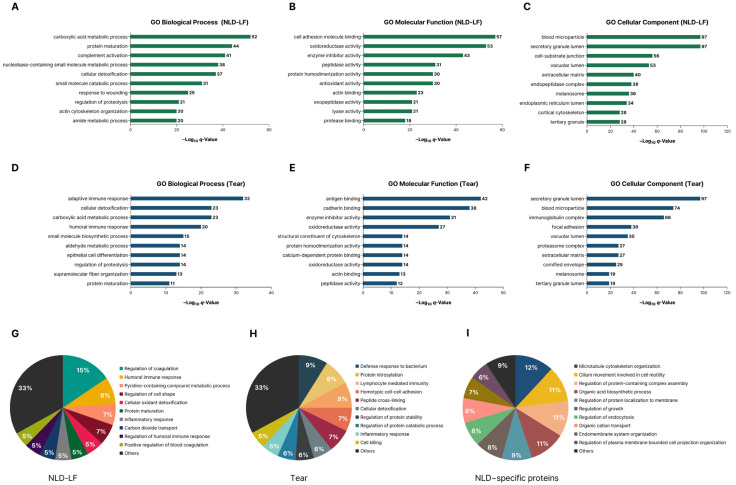
Functional enrichment analysis of NLD-LF and tear proteomes. Gene Ontology (GO) enrichment analysis was performed using Metascape across the Biological Process (BP), Molecular Function (MF), and Cellular Component (CC) categories. Terms with *p* < 0.01, a minimum count of 3, and an enrichment factor > 1.5 were considered significant. (**A**–**F**) Bar graphs show the top 10 enriched GO terms (BP, MF, and CC) ranked by −log_10_(q) values for the all identified proteins in NLD-LF and tear, respectively. (**G**–**I**) Pie graphs illustrate the top 10 enriched GO Biological Process (GO-BP) terms identified from the top proteins in NLD–LF, tear, and NLD-specific proteins, visualized based on gene count.

Gene Ontology (GO) enrichment analysis was performed on all identified proteins from NLD-LF and tear fluid across the biological process (BP), molecular function (MF), and cellular component (CC) categories (Figure 2A–F; Table S2).

GO-BP enrichment analysis showed that NLD-LF proteins were predominantly associated with metabolic and complement-related processes, including carboxylic acid metabolic process, protein maturation, complement activation, and cellular detoxification. Tear fluid, in contrast, was enriched for immune- and epithelium-related terms such as adaptive immune response, humoral immune response, and epithelial cell differentiation.

GO-MF enrichment analysis showed that NLD-LF proteins were enriched in terms related to molecular binding and enzymatic regulation, including cell adhesion molecule binding, oxidoreductase activity, enzyme inhibitor activity, and peptidase activity. Tear fluid was enriched for immune- and adhesion-related functions such as antigen binding, cadherin binding, enzyme inhibitor activity, and oxidoreductase activity.

GO-CC enrichment analysis revealed that secretory granule lumen and blood microparticle were among the most enriched terms in both NLD-LF and tear fluid. NLD-LF showed enrichment for terms such as cell–substrate junction and vacuolar lumen, whereas tear fluid was enriched for components including the immunoglobulin complex and cornified envelope.

Based on the overall trends observed in the entire protein datasets, a subset of the top 100 most abundant proteins (and 91 proteins for the NLD-specific proteome) was selected for further analysis. GO-BP enrichment analysis was applied to these proteins to evaluate their functional trends.

In NLD-LF, enriched GO-BP terms included regulation of coagulation and humoral immune response, with regulation of cell shape, protein maturation, and cellular oxidant detoxification. (Figure 2G). Tear fluid showed enrichment for defense response to bacterium and lymphocyte-mediated immunity. Additional terms such as protein nitrosylation, regulation of protein stability, regulation of protein catabolic process, homotypic cell–cell adhesion, and peptide cross-linking were also identified (Figure 2H). For the NLD-specific proteome, enriched terms included microtubule cytoskeleton organization, cilium movement involved in cell motility, and regulation of protein-containing complex assembly. Enrichment was also observed for membrane-associated processes such as regulation of protein localization to membrane, regulation of endocytosis, and regulation of plasma membrane–bounded cell projection organization (Figure 2I).

### 2.5. Comparative Analysis of Differentially Expressed Proteins Between Mucinous and Membranous Subtypes in NLD-LF

**Figure 3:**
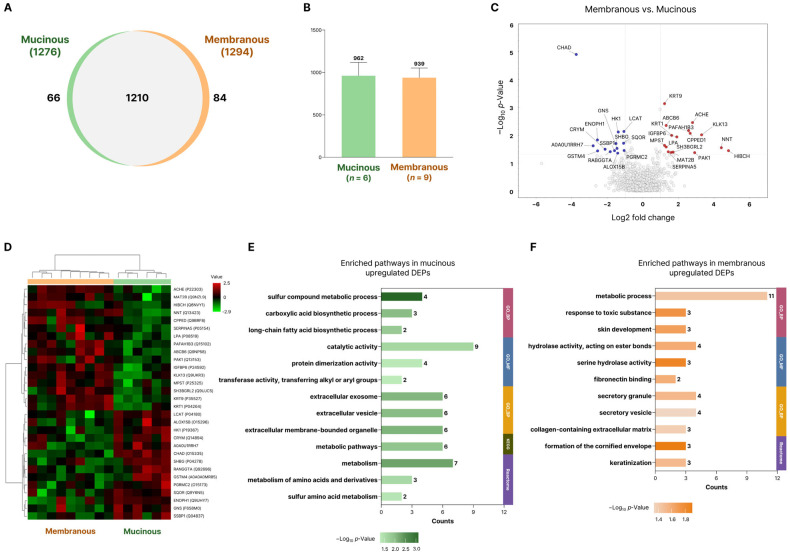
Comparative analysis of differentially expressed proteins (DEPs) between mucinous and membranous subtypes in NLD-LF. (**A**) Comparison of identified proteins between mucinous and membranous subtypes. (**B**) Comparison of the number of average identified proteins in mucinous and membranous subtypes. (**C**) Volcano plot illustrating differentially expressed proteins (DEPs) between the mucinous and membranous subtypes. Red and blue dots indicate significantly upregulated proteins in each subtype, defined by a |log2 fold change| > 1 and *p*-Value < 0.05. (**D**) Hierarchical clustering of the normalized abundances of DEPs. (**E**,**F**) Bar graphs illustrate GO, KEGG, and Reactome enrichment analyses of upregulated proteins in the mucinous (left) and membranous subtypes (right), performed using DAVID to identify significantly enriched biological functions in each subtype (*p*-Value < 0.05).

Protein expression profiles were compared between the mucinous (*n* = 6) and membranous (*n* = 9) NLD-LF groups. A total of 1210 proteins were commonly identified in both subtypes, with 1276 proteins detected in the mucinous subtype and 1294 proteins in the membranous subtype (Figure 3A). The average number of identified proteins per sample did not differ significantly between the two subtypes, indicating overall similarity in global protein composition (Figure 3B). Differentially expressed proteins (DEPs) were subsequently identified to assess functional differences between the two subtypes.

A comparison of protein expression between the membranous and mucinous NLD-LF groups identified a total of 30 differentially expressed proteins (DEPs) (Figure 3C,D; Table S3). Among these, 16 proteins showed higher expression in the membranous subtype, while 14 proteins were more highly expressed in the mucinous subtype. Although overall expression differences between the two subtypes were modest, distinct changes in specific proteins were observed and subsequently used for functional analysis.

Functional enrichment analysis of the DEPs revealed that proteins with higher expression in the mucinous subtype were enriched in metabolic pathways such as sulfur compound metabolic process, carboxylic acid biosynthetic process, and long-chain fatty acid biosynthetic process, as well as terms related to extracellular vesicles and other extracellular organelles (Figure 3E). Proteins upregulated in the membranous subtype were associated with pathways including metabolic process, skin development, and response to toxic substance, and were also enriched for extracellular matrix– and cornification-related terms such as fibronectin binding, collagen-containing extracellular matrix, and formation of the cornified envelope (Figure 3F).

### 2.6. Selection of Subtype-Specific Protein Candidates Distinguishing Mucinous and Membranous NLD-LF

**Figure 4:**
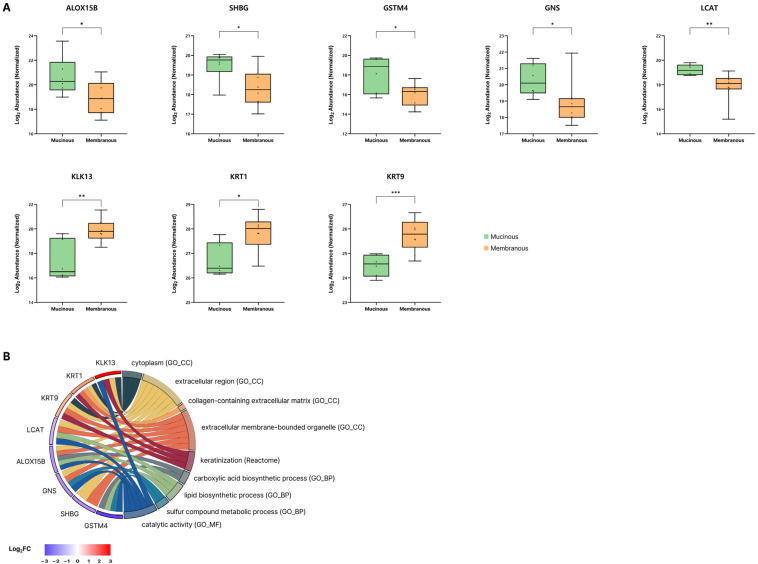
Functional analysis of subtype-specific proteins in NLD-LF. (**A**) Box plots showing the normalized abundance of polyunsaturated fatty acid lipoxygenase ALOX15B (*ALOX15B*), glutathione transferase (*GSTM4*), sex hormone-binding globulin (*SHBG*), N-acetylglucosamine-6-sulfatase (*GNS*), lecithin–cholesterol acyltransferase (*LCAT*), kallikrein-13 (*KLK13*), keratin type II cytoskeletal 1 (*KRT1*), and keratin type I cytoskeletal 9 (*KRT9*) as subtype-specific proteins for distinguishing between the mucinous and membranous subtypes. Statistical significance was indicated as * *p*-Value < 0.05, ** *p*-Value < 0.01, *** *p*-Value < 0.001. (**B**) Chord diagram illustrating the associations between subtype-specific proteins and biologically meaningful functional terms derived from GO and Reactome pathways, with color coding representing log2 fold-change (mucinous vs. membranous subtypes).

Based on the functional enrichment analysis of the DEPs, five candidate proteins distinguishing the mucinous and membranous subtypes were identified (Figure 4A). Polyunsaturated fatty acid lipoxygenase (*ALOX15B*), glutathione transferase (*GSTM4*), and sex hormone-binding globulin (*SHBG*) showed higher expression in the mucinous subtype, whereas kallikrein-13 (*KLK13*), and keratin type II cytoskeletal 1 (*KRT*) were more highly expressed in the membranous subtype. The major GO terms associated with these proteins are summarized in Figure 4B.

## 3. Discussion

To better understand the pathophysiology of PANDO, several studies have previously focused on analyzing tear components. However, this study demonstrated that the protein compositions of tears and NLD-LF differ substantially. Notably, whereas 68.8% of tear proteins were also detected in the NLD-LF, only 39.3% of NLD proteins overlapped with those found in tears. This asymmetric pattern suggests that the NLD environment harbors a distinct set of proteins that are not merely derived from the tear but are likely produced or modified within the nasolacrimal duct.

The lavage fluids obtained from patients with membranous and mucinous types of nasolacrimal duct obstruction shared majority of their protein components and exhibited similar proteomic profiles. Nevertheless, we observed distinct patterns of protein expression between the two obstruction types, suggesting that their underlying pathophysiological mechanisms may differ. To further characterize these subtype-specific differences within NLD-LF, differentially expressed proteins identified between the membranous and mucinous subtypes were subjected to functional enrichment analysis, suggesting subtype-related functional differences.

Specifically, membranous obstruction may be more closely associated with pathways related to fibrotic remodeling and epithelial barrier alterations, whereas mucinous obstruction may reflect enhanced secretory activity. Although the number of identified DEPs was limited, these subtype-specific proteins suggest potential heterogeneity within PANDO and emphasize the importance of considering obstruction type and their corresponding pathogenesis.

In the membranous subtype, the Reactome Keratinization pathway showed enrichment based on the upregulation of *KLK13*, *KRT1*, and *KRT9*. This pathway represents the canonical process of keratinocyte differentiation and cornification, in which epithelial cells progressively accumulate keratin intermediate filaments and cornified envelope proteins to reinforce the barrier, while proteases such as kallikreins regulate desquamation and turnover of the keratinized layer [8].

*KRT1* and *KRT9* are structural keratins expressed in differentiated keratinocytes that reinforce the epithelial barrier through intermediate filament assembly [9,10]. Their increased abundance may reflect squamous-like epithelial remodeling characterized by enhanced cornification and barrier reinforcement within the nasolacrimal duct mucosa. In parallel, *KLK13*, a serine protease implicated in epithelial barrier regulation and desquamation via cleavage of adhesion structures such as corneodesmosomes, was elevated, suggesting a possible association with keratinization-related processes [11,12,13]. While the number of DEPs is limited relative to the broader pathway, the coordinated increase in *KRT1*, *KRT9*, and *KLK13* is consistent with a remodeling process in which the duct epithelium becomes more keratinized, potentially contributing to the accumulation of keratinized debris and recurrent mechanical obstruction.

In the mucinous subtype, the upregulated proteins *GSTM4*, *LCAT*, and *ALOX15B* were prominently enriched in pathways related to lipid synthesis, suggesting that alterations in luminal lipid metabolism contribute to the formation of mucin-rich obstruction. *GSTM4*, which is involved in the conjugation and detoxification of lipid peroxidation–derived electrophilic aldehydes, is typically upregulated under conditions of oxidative lipid stress [14,15]. Its elevated implies increased turnover of oxidized lipids within the nasolacrimal duct lumen. Similarly, *LCAT* plays a key role in remodeling phospholipid membranes and lipoprotein-like structures [16,17].

Within this lipid metabolic framework, *ALOX15B* serves as an upstream generator of oxidized polyunsaturated fatty acid derivatives, including 15-hydroxyeicosatetraenoic acid (15-HETE) and oxidized phospholipids. Although direct evidence linking *ALOX15B* to mucin regulation is limited, extensive prior work demonstrates that its related isoform *ALOX15* promotes goblet cell differentiation and *MUC5AC* upregulation in airway epithelium through oxidized phospholipids such as 15-HETE-PE [18,19]. Collectively, these findings suggest a biological link between lipid oxidation pathways and mucin regulation, raising the possibility that *ALOX15B* may contribute to a similar lipid-mediated mucinogenic response in the nasolacrimal duct.

Beyond lipid biosynthesis, enrichment of the carboxylic acid metabolic process indicates that the accumulation of fatty acids and oxidized derivatives may alter the physical properties of mucin. Carboxylic acids are known to interact with gel-forming mucins and modify their viscoelastic behavior. In airway and gastrointestinal epithelia, increases in lipid content within the mucus layer enhance hydrophobic interactions, mucin packing density, and gel stiffness, ultimately leading to impaired mucus transport and luminal stasis [20,21,22]. These physicochemical effects provide a plausible mechanism by which altered carboxylic acid metabolism contributes to the formation of dense, adhesive mucinous material in the nasolacrimal duct.

In parallel, the mucinous subtype demonstrated significant enrichment of the sulfur compound metabolic process, involving *ENOPH1*, *GNS*, and *GSTM4*. Sulfur metabolism is a key determinant of mucin structure and rheology, as sulfation of mucin O-glycans directly regulates mucus viscoelasticity, hydration, and barrier properties. Increased sulfation enhances mucin charge density and polymer–polymer interactions, resulting in a more cohesive and less soluble gel network [23,24]. *GNS*, a lysosomal sulfatase, modulates the sulfation state of glycosaminoglycans and mucin-associated glycans, and altered *GNS* expression may reflect increased turnover or remodeling of sulfated substrates within the obstructed lumen. In addition, Oxidative conditions promote disulfide bond formation, increasing mucin cross-linking and gel stiffness—changes strongly associated with mucus plugging in airway disease [25,26]. The upregulation of *GSTM4*, a glutathione-related detoxification enzyme, suggests a shift toward a more oxidizing luminal environment, which would further reinforce mucin gel structure and reduce its degradability [14,26].

These findings indicate that mucinous obstruction is driven by coordinated alterations in lipid, carboxylic acid, and sulfur metabolic pathways. These pathways converge to enhance mucin–lipid interactions, increase glycan sulfation, and promote redox-dependent mucin cross-linking, ultimately leading to the secretory obstruction of nasolacrimal duct.

Although the overall protein composition of the membranous and mucinous subtypes was largely comparable and the number of identified differentially expressed proteins (DEPs) was limited, pathway-level analyses revealed differences in protein expression patterns associated with their underlying pathophysiology. This may suggest that, despite broadly similar proteomic profiles, the two subtypes involve partially distinct molecular processes related to obstruction.

## 4. Materials and Methods

### 4.1. Subjects

This study included patients diagnosed with PANDO who underwent dacryoendoscopy-assisted silicone tube intubation of the nasolacrimal duct at Bundang CHA Hospital from August to December 2023. The diagnosis of PANDO was made in patients who reported epiphora with a Munk score of 2 or higher, showed reflux during syringing, and demonstrated dacryocystographic evidence of obstruction, including significant duct narrowing, complete obstruction, or secondary dilation of the NLD. Dacryoendoscopic evaluation was used to classify the obstruction as either membranous or mucous.

### 4.2. Sample Collection of NLD and Tear Fluids

Tear fluid was obtained from the lower conjunctival sac using a sterile Weck-Cel ophthalmic sponge (BVI Medical, Waltham, MA, USA). To avoid alterations in tear protein composition, no topical anesthetic agents were applied prior to sampling. After collection, only the sponge tip was detached and placed into a sterile conical tube, followed by storage at −80 °C until further protein analysis. NLD lavage fluids were collected during dacryoendoscopic recanalization. An 18-gauge sheath was fitted over the dacryoendoscope, and NLD recanalization was performed. 2 mL of normal saline was gently irrigated through the sheath, which was collected simultaneously using a suction tip. The outflowing fluid was simultaneously collected using a suction tip placed at the inferior meatus, and the irrigated sample was transferred into a sterile suction bottle. All samples were immediately stored at −80 °C prior to proteomic analysis.

### 4.3. Protein Extraction and Digestion

Tear samples were lysed in 8 M urea–0.1 M Tris-HCl buffer (pH 8.5), and the protein extract eluted from the sponge was collected using a 100 kDa Amicon^®^ Ultra centrifugal filter (Millipore, Burlington, MA, USA). For NLD lavage fluids, highly abundant plasma-derived proteins were removed using the High Select™ Top14 Abundant Protein Depletion Resin (Thermo Fisher Scientific, Waltham, MA, USA) to minimize potential interference caused by blood contamination during collection. Protein concentrations were determined using the Pierce™ BCA Protein Assay Kit (Thermo Fisher Scientific, Waltham, MA, USA). From each sample, 45 µg of total protein was subjected to automated filter-aided sample preparation (aFASP). Enzymatic digestion was performed on a liquid-handling robotic platform (Agilent Technologies, Santa Clara, CA, USA) operated via VWorks software (v11.4.0.1233), according to a previously established protocol [27]. In brief, proteins were reduced with 5 mM tris(2-carboxyethyl)phosphine (TCEP) (Thermo Fisher Scientific, Waltham, MA, USA) at 33 °C for 30 min in a 96-well filter plate, followed by alkylation with 50 mM iodoacetamide (Sigma-Aldrich, St. Louis, MO, USA) at room temperature for 1 h in the dark. After sequential washing steps with lysis buffer and 50 mM ammonium bicarbonate, digestion was carried out using sequencing-grade modified trypsin (Promega, Madison, WI, USA) at a 1:50 (*w*/*w*) enzyme-to-protein ratio for 18 h at 37 °C. After tryptic digestion, peptides were recovered by centrifugation and dried in a SpeedVac system (Gyrozen Co., Ltd., Seoul, Republic of Korea). The dried peptides were resuspended in 0.1% trifluoroacetic acid and desalted using C18 spin columns (Harvard Apparatus, Holliston, MA, USA). The desalted peptides were stored at −20 °C until LC–MS/MS analysis.

### 4.4. LC–MS/MS Analysis

Peptide samples were reconstituted in 0.1% formic acid in water and analyzed using a Q Exactive Orbitrap hybrid mass spectrometer (Thermo Fisher Scientific, Waltham, MA, USA) coupled to an Ultimate 3000 nanoLC system (Thermo Fisher Scientific, Waltham, MA, USA). For each sample, 500 ng of peptides was loaded onto an Acclaim™ PepMap™ 100 C18 trap column (3 μm, 75 μm × 2 cm) and separated on an EASY-Spray™ PepMap™ RSLC C18 analytical column (2 μm, 75 μm × 50 cm). The mobile phases comprised 0.1% formic acid in water (A) and 0.1% formic acid in 80% acetonitrile (B) (JT Baker, Philipsburg, NJ, USA), operated at a constant flow rate of 300 nL/min. The gradient of mobile phase was as follows: 4% solvent B in 14 min, 4–15% solvent B in 61 min, 15–28% solvent B in 50 min, 28–40% solvent B in 20 min, 40–96% solvent B in 2 min, holding at 96% of solvent B in 13 min, 96–4% solvent B in 1 min, and 4% solvent B for 24 min. Data-dependent acquisition (DDA) was performed with selection of the top 10 most intense precursor ions for fragmentation. Full MS scans were acquired at a resolution of 70,000 (at m/z 400), followed by MS/MS scans at a resolution of 17,500. The mass scan range was set to 400–2000 m/z. Precursor ion fragmentation was conducted at 27% normalized collision energy (NCE), with dynamic exclusion set to 30 s.

### 4.5. Data Processing and Statistical Analysis

Raw MS/MS data were analyzed using Proteome Discoverer™ (ver. 3.1) (Thermo Fisher Scientific, Waltham, MA, USA) and searched against the UniProt Homo sapiens reference proteome (release 2022.05). Peptide identification was performed using the SEQUEST HT search engine within the consensus workflow, with peptide-spectrum matches validated through built-in quality control criteria. Database searching was conducted with a precursor mass tolerance of 10 ppm and a fragment mass tolerance of 0.02 Da, allowing up to two missed cleavages for tryptic peptides. Carbamidomethylation of cysteine (+57.021 Da) was specified as a fixed modification, whereas oxidation of methionine (+15.995 Da), N-terminal acetylation (+42.011 Da), and N-terminal carbamylation (+43.006 Da) were treated as variable modifications. Identifications were filtered to achieve a false discovery rate (FDR) of less than 1% at the peptide level, and only peptides comprising at least six amino acids were retained. Protein abundances were normalized using the Total Peptide Abundance method, in which the total peptide signal of each LC–MS/MS run was scaled to match that of the run with the highest total abundance, thereby reducing technical variation across samples. Missing values were imputed using Low Abundance Resampling, whereby replacement values were randomly drawn from the lower 5% of detected intensity values. Fold change was calculated using the Protein Abundance Based approach, where protein ratios were directly derived from grouped protein abundances. *p*-Values were calculated using a background-based *t*-test, in which the abundance ratio of each protein was assessed relative to the overall distribution of quantified protein ratios in the dataset. Enrichment analyses for Gene Ontology (GO), KEGG pathways, and Reactome pathways were conducted using Metascape v3.5.20250701 (https://metascape.org/, accessed on 10 November 2025) and DAVID 6.8 (https://davidbioinformatics.nih.gov/, accessed on 11 November 2025). Multivariate analyses and heatmap visualization were performed with MetaboAnalyst 6.0 (https://www.metaboanalyst.ca/, accessed on 13 October 2025) [28,29].

## 5. Conclusions

This study provides the first comprehensive proteomic characterization of nasolacrimal duct lavage fluid in patients with primary acquired nasolacrimal duct obstruction. By directly analyzing the biochemical environment within the nasolacrimal duct, we identified a distinct NLD-specific proteome that differs substantially from tear fluid and reflects local metabolic and structural processes. Although the membranous and mucinous subtypes shared broadly similar protein compositions, their differentially expressed proteins revealed divergent biological pathways associated with obstruction. The membranous subtype was characterized by keratinization-related remodeling, whereas the mucinous subtype showed metabolic alterations that promote mucin–lipid interactions, glycan sulfation, and redox-dependent mucin cross-linking. As an exploratory proteomic analysis, these findings suggest potential heterogeneity within PANDO and indicate that obstruction may involve subtype-related molecular differences rather than a single uniform pathway. However, further validation in larger cohorts, particularly in studies including healthy control groups, will be required to confirm these observations and to better define their potential clinical relevance.

## Figures and Tables

**Table 1:** Patient demographics.

	Membranous (*n* = 9)	Mucinous (*n* = 6)	Total (*n* = 15)	*p*-Value
Age (yrs)	62.9 ± 16.4	67.3 ± 7.6	64.7 ± 13.4	0.493
Sex (M:F)	4:5	2:4	6:9	0.667
Side (OD:OS)	3:6	5:1	8:7	0.057
Tear meniscus height (μm)	346.2 ± 134.7	331.7 ± 100.0	340.4 ± 118.3	0.814
Syringing				
Partially passed (*n*, %)	8 (88.9%)	6 (100%)	14 (93.3%)	0.398
Not passed (*n*, %)	1 (11.1%)	0 (0%)	1 (6.7%)	
Dacryocystography				
Beaded	4 (44.4%)	3 (50%)	7 (46.7%)	0.418
Narrowing	1 (11.1%)	2 (33.3%)	3 (20%)	
Obstruction	4 (44.4%)	1 (16.7%)	5 (33.3%)	

**Table 2:** Top 10 abundant identified proteins in NLD-LF, tear, and NLD-specific proteomes.

	Accession	Gene Name	Description	Unique Peptide
NLD-LF	P69905	*HBA1*	Hemoglobin subunit alpha	128
	P68871	*HBB*	Hemoglobin subunit beta	56
	P02768	*ALB*	Albumin	85
	P00915	*CA1*	Carbonic anhydrase 1	36
	P01024	*C3*	Complement C3	162
	P02042	*HBD*	Hemoglobin subunit delta	26
	P32119	*PRDX2*	Peroxiredoxin-2	32
	P04040	*CAT*	Catalase	64
	P02790	*HPX*	Hemopexin	59
	P98088	*MUC5AC*	Mucin-5AC	153
Tear	P61626	*LYZ*	Lysozyme C	71
	P02788	*LTF*	Lactotransferrin	164
	P31025	*LCN1*	Lipocalin-1	47
	P04264	*KRT1*	Keratin, type II cytoskeletal 1	83
	P02768	*ALB*	Albumin	99
	P01876	*IGHA1*	Immunoglobulin heavy constant alpha 1	25
	A0A1B0GVI3	*KRT10*	Keratin, type I cytoskeletal 10	63
	P35527	*KRT9*	Keratin, type I cytoskeletal 9	92
	P01833	*PIGR*	Polymeric immunoglobulin receptor	71
	Q9GZZ8	*LACRT*	Extracellular glycoprotein lacritin	16
NLD-specific	A0A2R8Y7X9	-	GLOBIN domain-containing protein	1
	Q92902	*HPS1*	Hermansky-Pudlak syndrome 1 protein	1
	Q32Q12	*NME1-NME2*	Nucleoside diphosphate kinase	3
	Q5VX52	*SPATA1*	Spermatogenesis-associated protein 1	1
	P69892	*HBG2*	Hemoglobin subunit gamma-2	1
	Q71U36	*TUBA1A*	Tubulin alpha-1A chain	6
	Q96F07	*CYFIP2*	Cytoplasmic FMR1-interacting protein 2	2
	O60635	*TSPAN1*	Tetraspanin-1	2
	P16050	*ALOX15*	Polyunsaturated fatty acid lipoxygenase ALOX15	15
	P43353	*ALDH3B1*	Aldehyde dehydrogenase family 3 member B1	7

## Data Availability

The mass spectrometry proteomics data are available on the ProteomeXchange Consortium via the PRIDE partner repository with the dataset identifier PXD072516.

## Supplementary Materials

The following supporting information can be downloaded at: https://www.mdpi.com/article/10.3390/ijms27062616/s1.

## Abbreviations

The following abbreviations are used in this manuscript:

PANDO	primary acquired nasolacrimal duct obstruction
NLD	nasolacrimal duct
NLD-LF	nasolacrimal duct lavage fluid
DEPs	differentially expressed proteins
PCA	principal component analysis
